# Obstetric and Perinatal Outcomes of Singleton Births Following Single- vs Double-Embryo Transfer in Sweden

**DOI:** 10.1001/jamapediatrics.2022.4787

**Published:** 2022-12-05

**Authors:** Kenny A. Rodriguez-Wallberg, Arturo Reyes Palomares, Hanna P. Nilsson, Anna Sara Oberg, Frida Lundberg

**Affiliations:** 1 Department of Oncology-Pathology, Laboratory of Translational Fertility Preservation, Karolinska Institutet, Stockholm, Sweden; 2 Division of Gynecology and Reproduction, Department of Reproductive Medicine, Karolinska University Hospital, Stockholm, Sweden; 3 Department of Medical Epidemiology and Biostatistics, Karolinska Institutet, Stockholm, Sweden

## Abstract

**Importance:**

Pregnancies resulting from assisted reproductive technology are associated with an increased risk of adverse perinatal outcomes compared with those following natural conception. Previous studies have shown an association of pregnancies resulting from transfer of multiple embryos with these negative findings.

**Objective:**

To determine the risk for adverse outcomes in singletons conceived through assisted reproduction using double-embryo transfer (DET) vs single-embryo transfer (SET).

**Design, Setting, and Participants:**

This cohort study used data from women who achieved singleton deliveries after SET or DET in Sweden between 2007 and 2017 as recorded in the National Quality Registry for Assisted Reproduction. All embryo transfers, at cleavage or blastocyst stage, replaced in fresh or frozen treatment cycles were included. Data on obstetric and neonatal outcomes were retrieved by linkage to the National Medical Birth Register. Naturally conceived singletons were included as a reference group. Data were analyzed between September 2021 and August 2022.

**Exposures:**

Double-embryo transfer leading to singleton birth.

**Main Outcomes and Measures:**

Relative risk ratios or odds ratios (ORs) and absolute risk differences (ARDs) in percentage points with 95% CIs were calculated for obstetric and perinatal outcomes in singleton births conceived using DET vs SET.

**Results:**

Among 1 115 863 singleton births, 30 713 singletons were born after SET and 5123 after DET. A higher risk of neonatal death was found in singletons after DET vs SET (OR, 2.67 [95% CI, 1.28-5.55]; ARD, 0.2 percentage points [95% CI, 0.0-0.4 percentage points]). In frozen embryo transfers, DET was associated with a higher risk of low birth weight (OR, 1.64 [95% CI, 1.19-2.25]; ARD, 2.0 percentage points [95% CI, 0.5-3.5 percentage points]). Among blastocyst transfers, DET was associated with very preterm birth (relative risk ratio, 2.64 [95% CI, 1.50-4.63]; ARD, 1.8 percentage points [95% CI, 0.3-3.4 percentage points]) and low birth weight (OR, 1.83 [95% CI, 1.29-2.60]; ARD, 3.2 percentage points [95% CI, 0.9-5.5 percentage points]).

**Conclusions and Relevance:**

These results indicate a higher risk of adverse outcomes following DET, even when the result is a singleton birth, vs singletons born after SET. Adverse outcomes were mainly observed in singletons following DET using frozen embryos and blastocysts.

## Key Points

**Question:**

Is double-embryo transfer (DET) associated with a risk of adverse outcomes in singleton births conceived through assisted reproduction?

**Findings:**

In this cohort study of 1 115 863 singleton births, a higher risk of preterm delivery and low birth weight followed DET vs single-embryo transfer (SET) when using cryopreserved embryos or blastocysts. Although rare, there was also a higher risk of neonatal death in DET vs SET singletons.

**Meaning:**

Results suggest a higher risk of adverse outcomes in singleton births after DET vs SET, especially when using frozen embryos and blastocysts.

## Teaser

This cohort study evaluates adverse outcomes of singletons conceived through assisted reproduction using single-embryo transfer (SET) vs double-embryo transfer (DET) in Sweden.

## Introduction

To improve the safety of assisted reproductive technology (ART) and reduce obstetric risks, some countries have restrictions on the number of transferred embryos and actively promote single-embryo transfer (SET), which efficiently reduces high-risk twin or higher-order gestations.^1^ The need to decrease the number of twin pregnancies is motivated by health economy; maternal health risks; clinical complications; and increased adverse neonatal and perinatal outcomes, such as low birth weight, prematurity, and perinatal morbidity after higher-order gestations.^1^ In some countries, legislation encourages elective SET, whereas in a majority of countries, the transfer of multiple embryos is not regulated.^7^ The use of elective SET is supported by studies from centers performing mostly SET, showing a significant decrease of twin pregnancies but no impairment of cumulative birth rates,^8^ and by studies in which subsequent frozen embryo transfers have been shown to provide equal pregnancy rates at a lower risk for adverse outcomes.^5^

Double-embryo transfer (DET) is not only often desired by patients but also more commonly used in cases in which older age, previous implantation failure, poor embryo quality, or other unfavorable conditions reduce the chance of pregnancy.^1^ Lower fertility potential reduces the likelihood of DET resulting in multiple gestation.^5^ Thus, many patients undergoing DET have a singleton pregnancy.

Compared with naturally conceived births, ART is associated with adverse neonatal outcomes.^18^ A meta-analysis from 2022 reported a lower risk for poor outcomes after SET vs DET, although it mainly focused on live birth rate and the risk of multiple gestation.^16^ Large in-depth studies on the obstetric outcomes of singleton pregnancies comparing DET with SET are currently lacking. The aim of our study was to investigate whether obstetric and neonatal adverse outcomes of singletons born through ART differed with the use of SET vs DET.

## Methods

This cohort study used Swedish national population-based registries. All live births in Sweden between July 1, 2007, and December 31, 2017, were identified in the National Medical Birth Register (MBR). Assisted reproductive technology births were identified in the National Quality Registry for Assisted Reproduction (Q-IVF). All ART cycles leading to live birth, using either fresh or frozen embryo transfer, were linked to the MBR data using the unique personal identification number of the mothers and the date of birth of the children (±31 days). The regional ethics committee in Stockholm approved the study. Informed consent exemption was granted because this was a register-based study. This study followed the Strengthening the Reporting of Observational Studies in Epidemiology (STROBE) reporting guideline.

We excluded births in the Q-IVF with no match in the MBR, multiple births, births registered as ART in the MBR but not identified in the Q-IVF, ART births registered as neither SET nor DET, and ART births following oocyte donation treatments. Furthermore, singleton births with genetic or chromosomal abnormalities registered in the MBR were excluded, as were births to mothers younger than 19 or older than 47 years because no one younger or older than these ages had undergone ART (Figure).

**Figure.**
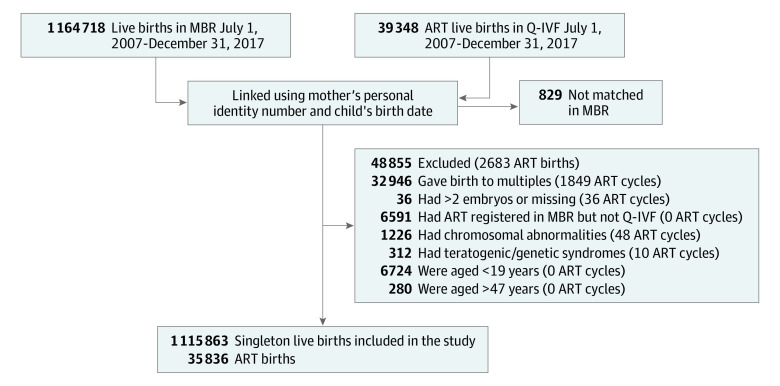
Study Flow Diagram ART indicates assisted reproductive technology; MBR, National Medical Birth Register; and Q-IVF, National Quality Registry for Assisted Reproduction.

Data extracted from the MBR included birth order of the child, year of delivery, maternal age, prepregnancy body mass index (BMI; calculated as weight in kilograms divided by height in meters squared), self-reported smoking during pregnancy, and complications specific to multiple gestation. The mother’s education level at the year of birth was obtained from the Longitudinal Integrated Database for Health Insurance and Labour Market Studies. Infertility-related diagnoses before delivery were identified in the National Patient Register. For ART births, information from the Q-IVF included number of embryos transferred, stimulation protocol, fertilization method (in vitro fertilization [IVF] or intracytoplasmic sperm injection [ICSI]), embryo culture duration, fresh or frozen embryo transfer, use of preimplantation genetic diagnosis, number of gestational sacs at ultrasound in week 7, information on a second fetus (ie, spontaneous or induced fetal reduction), and total number of fresh stimulation cycles before delivery. Pregnancies with 2 gestational sacs, information on a second fetus, or complications specific to multiple gestation that ended in singleton births were categorized as possible vanishing twins. From *International Classification of Diseases, Tenth Revision* (*ICD-10*) codes, female infertility diagnoses (N97), diagnosis of ovulatory disturbance (E28 or N97.0) or endometriosis (N80 or N97.8D), and hypothyroidism (E01-E03) before delivery were identified in the National Patient Register.

The following pregnancy and delivery outcomes were identified in the MBR: gestational hypertension (*ICD-10* codes O13-O15), preeclampsia (*ICD-10* codes O14-O15), gestational diabetes (*ICD-10* code O24), placental abruption (*ICD-10* code O45), prelabor rupture of membranes (*ICD-10* code O42), bleeding during pregnancy (*ICD-10* codes O20, O46, and O67), mode of delivery, induced delivery, infant death within 27 days of delivery, gestational age, birth weight, Apgar score at 5 minutes, congenital malformations, and sex of the baby. Birth weight for gestational age (BWGA) was calculated according to Swedish standard curves.^19^ Congenital malformations were categorized as major or minor malformations according to the European Network of Population-Based Registries for the Epidemiological Surveillance of Congenital Anomalies (eTable 1 in the Supplement).

### Statistical Analysis

Analyses were conducted between September 2021 and August 2022. Pregnancy and birth outcomes were compared between singleton births following DET and SET. Logistic regression was used to estimate odds ratios (ORs) for binary outcomes, whereas multinomial logistic regression was used to estimate relative risk ratios (RRRs) for categorical outcomes, with 95% CIs. The adjusted models included maternal age, birth order, delivery year, maternal country of birth, previous spontaneous abortions, extrauterine pregnancies, and number of stimulation cycles. Adjusted absolute risks in percent and absolute risk differences (ARDs) in percentage points with 95% CIs were calculated from the models using the margins command in Stata, version 17.0/BE (StataCorp LLC). Births with missing outcome information (<0.5% for any outcome) were excluded from the models for that outcome. For reference, the outcomes were also compared between singleton births from SET or DET with those of naturally conceived singleton pregnancies. To investigate the influence of extended embryo culture and cryopreservation of embryos, we stratified the cohort by fresh or frozen cycles and by cleavage stage or blastocyst transfer and tested for interactions using likelihood ratio tests. In frozen cycles separately, we also adjusted for time since embryo cryopreservation and the type of endometrial preparation (natural cycle or exogenous estrogen and progesterone). Pregnancy and birth outcomes were also compared between DET and SET after exclusion of possible twin pregnancies and among first births only. We further adjusted for fertilization method, maternal education, BMI, and smoking during pregnancy using multiple imputation with chained equations to account for missing values of these covariates.

Data management was performed using SAS, version 9.4 (SAS Institute Inc), and analyses were performed using Stata, version 17.0/BE. All tests were 2-sided with a significance level of 5%.

## Results

The final study population consisted of 1 115 863 singleton births, of which 35 836 resulted from ART treatment using either SET (n = 30 713) or DET (n = 5123). The demographic characteristics of the cohort are presented in Table 1. Mean (SD) maternal age at delivery was higher among women undergoing DET (36.2 [4.19] years) compared with women undergoing SET (33.4 [4.19] years). Double-embryo transfer was more common in the earliest cohort years, 2007 through 2010 (DET, 1968 [38.4%]; SET, 7504 [24.4%]). There were no major differences between the 2 study groups with regard to parity, country of birth, educational level, family situation, BMI, smoking habits, maternal medical conditions, infertility diagnosis, and previous stillbirths. Vanishing twin syndrome, previous spontaneous abortions, and previous extrauterine pregnancies were more common among women undergoing DET. With regard to the method of assisted reproduction, a higher percentage of the women in the DET group underwent ICSI (DET, 2600 [50.8%]; SET, 12 547 [40.9%]), had embryos transferred at the cleavage stage (DET, 4602 [89.8%]; SET, [19 737] 64.3%), and more often had fresh embryo transfers (DET, 4211 [82.2%]; SET, 19 771 [64.4%]). In frozen cycles, artificial endometrial preparation was more common in SET than in DET (DET, 503 [55.2%]; SET, 7234 [66.1%]), whereas the majority of embryo transfers were performed within 1 year of cryopreservation in both groups (DET, 586 [64.3%]; SET, 7294 [66.7%]).

**Table 1.** Description of Cohort

Characteristic	No. (%)		
	DET (n = 5123)	SET (n = 30 713)	Natural conception (n = 1 080 027)
Maternal age, y			
19-24	22 (0.4)	587 (1.9)	151 439 (14.0)
25-29	376 (7.3)	5019 (16.3)	334 434 (31.0)
30-34	1258 (24.6)	12 181 (39.7)	367 616 (34.0)
35-39	2203 (43.0)	10 843 (35.3)	186 549 (17.3)
40-47	1264 (24.7)	2083 (6.8)	39 989 (3.7)
Birth order			
First child	3396 (66.3)	20 404 (66.4)	461 734 (42.8)
Second child	1453 (28.4)	8859 (28.8)	403 299 (37.3)
Third or higher order	274 (5.3)	1450 (4.7)	214 994 (19.9)
Delivery year			
2007-2010	1968 (38.4)	7504 (24.4)	352 677 (32.7)
2011-2014	2136 (41.7)	11 770 (38.3)	412 077 (38.2)
2015-2017	1019 (19.9)	11 439 (37.2)	315 273 (29.2)
Maternal country of birth			
Nordic	4259 (83.1)	25 128 (81.8)	819 087 (75.8)
Non-Nordic	864 (16.9)	5585 (18.2)	260 940 (24.2)
Maternal education at year of delivery			
Compulsory school	207 (4.0)	1386 (4.5)	119 981 (11.1)
Secondary school	1440 (28.1)	8800 (28.7)	381 359 (35.3)
Higher education			
<3 y	719 (14.0)	4159 (13.5)	138 515 (12.8)
≥3 y	2688 (52.5)	15 883 (51.7)	368 321 (34.1)
Missing	69 (1.3)	485 (1.6)	71 851 (6.7)
Maternal family situation			
Cohabiting with father of child	4796 (93.6)	28 536 (92.9)	966 518 (89.5)
Single	13 (0.3)	54 (0.2)	20 484 (1.9)
Other	114 (2.2)	555 (1.8)	43 888 (4.1)
Missing	200 (3.9)	1568 (5.1)	49 134 (4.5)
Maternal BMI			
<18.5	74 (1.4)	612 (2.0)	25 452 (2.4)
18.5-24.9	3036 (59.3)	19 080 (62.1)	596 346 (55.2)
25.0-29.9	1410 (27.5)	7749 (25.2)	255 793 (23.7)
≥30	525 (10.2)	2935 (9.6)	132 035 (12.2)
Missing	78 (1.5)	337 (1.1)	70 401 (6.5)
Smoking during pregnancy			
No	4873 (95.1)	29 101 (94.8)	976 664 (90.4)
Yes	88 (1.7)	548 (1.8)	68 165 (6.3)
Missing	162 (3.2)	1064 (3.5)	35 198 (3.3)
Possible vanishing twin syndrome^a^	408 (8.0)	101 (0.3)	162 (0.0)
Previous spontaneous abortion	1841 (35.9)	8133 (26.5)	237 395 (22.0)
Previous extrauterine pregnancy	275 (5.4)	1254 (4.1)	13 520 (1.3)
Previous still birth	34 (0.7)	198 (0.6)	8119 (0.8)
Medical conditions			
Ovulatory disturbances^b^	771 (15.0)	5217 (17.0)	24 544 (2.3)
Endometriosis^b^	438 (8.5)	2448 (8.0)	9505 (0.9)
Hypothyroidism^b^	457 (8.9)	2654 (8.6)	36 023 (3.3)
Infertility diagnosis			
Anovulatory^b^	592 (11.6)	4128 (13.4)	10 838 (1.0)
Structural^b^	407 (7.9)	2393 (7.8)	2769 (0.3)
Due to endometriosis^b^	245 (4.8)	1339 (4.4)	1210 (0.1)
Male factor^b^	1254 (24.5)	7172 (23.4)	5565 (0.5)
Other factor^b^	680 (13.3)	3993 (13.0)	4538 (0.4)
Unexplained only	432 (16.0)	4133 (22.1)	4817 (18.4)
Unspecified only	1106 (29.1)	5918 (24.0)	30 228 (53.6)
No record of infertility in the NPR	1320 (25.8)	6048 (19.7)	1 023 550 (94.8)
Preimplantation genetic diagnosis	29 (0.6)	180 (0.6)	NA
Stimulation cycles before delivery			
1	1430 (27.9)	18 074 (58.8)	NA
2	1187 (23.2)	7526 (24.5)	NA
3	1106 (21.6)	3057 (10.0)	NA
≥4	1400 (27.3)	2056 (6.7)	NA
Fertilization method			
IVF	2253 (44.0)	16 016 (52.1)	NA
ICSI	2600 (50.8)	12 547 (40.9)	NA
Combination	213 (4.2)	1834 (6.0)	NA
Missing	57 (1.1)	316 (1.0)	NA
Embryo culture duration			
Cleavage stage	4602 (89.8)	19 737 (64.3)	NA
Blastocyst stage	495 (9.7)	10 896 (35.5)	NA
Missing	26 (0.5)	80 (0.3)	NA
Embryo transfer			
Fresh	4211 (82.2)	19 771 (64.4)	NA
Frozen	912 (17.8)	10 942 (35.6)	NA
Stimulation protocol in fresh cycles			
Agonist	1184 (28.1)	5133 (26.0)	NA
Antagonist	737 (17.5)	4364 (22.1)	NA
Missing	2290 (54.4)	10 274 (52.0)	NA
Endometrial preparation in frozen cycles			
Artificial cycle	503 (55.2)	7234 (66.1)	NA
Natural cycle	368 (40.4)	3507 (32.1)	NA
Missing	41 (4.5)	201 (1.8)	NA
Years since embryo freezing			
<1	586 (64.3)	7294 (66.7)	NA
1-2	209 (22.9)	2468 (22.6)	NA
≥3 y	117 (12.8)	1143 (10.4)	NA
Missing	0 (0.0)	37 (0.3)	NA

Abbreviations: BMI, body mass index (calculated as weight in kilograms divided by height in meters squared); DET, double-embryo transfer; ICSI, intracytoplasmic sperm injection; IVF, in vitro fertilization; MBR, National Medical Birth Register; NA, not applicable; NPR, National Patient Register; SET, single-embryo transfer.

^a^ Two gestational sacs at ultrasound or information on second fetus in the National Quality Registry for Assisted Reproduction or complications specific to multiple gestation (*International Classification of Diseases, Tenth Revision* code O31) in the MBR.

^b^ Not mutually exclusive.

### Risk Assessment for SET vs DET

In the full cohort of singletons conceived using SET or DET (Table 2), there were no significant differences in risk of gestational hypertension, preeclampsia, gestational diabetes, placental abruption, bleeding during pregnancy, prelabor rupture of membranes, delivery mode, or induction. The OR of neonatal mortality (infant death within 27 days) was higher in births following DET vs SET (OR, 2.67; 95% CI, 1.28-5.55). However, the adjusted absolute risk was 0.3% (95% CI, 0.1%-0.5%) in DET and 0.1% (95% CI, 0.1%-0.2%) in SET, and the ARD of 0.2 percentage points was not statistically significant (95% CI, 0.0-0.4 percentage points). No significant differences in gestational age, low birth weight, low Apgar score, BWGA, or congenital malformations were observed between SET and DET.

**Table 2.** Pregnancy and Birth Outcomes in Double-Embryo Transfer (DET) vs Single-Embryo Transfer (SET) Pregnancies

Outcome	AAR, % (95% CI)		ARD, percentage points (95% CI)	RRR or OR (95% CI)^a^
	DET	SET		
Gestational hypertension (yes vs no)	5.9 (5.2 to 6.6)	5.8 (5.5 to 6.1)	0.1 (−0.7 to 0.8)	1.02 (0.89 to 1.16)
Preeclampsia (yes vs no)	3.8 (3.3 to 4.3)	3.9 (3.7 to 4.2)	−0.1 (−0.7 to 0.5)	0.96 (0.82 to 1.14)
Gestational diabetes (yes vs no)	1.9 (1.5 to 2.3)	2.1 (2.0 to 2.3)	−0.2 (−0.6 to 0.2)	0.89 (0.71 to 1.11)
Bleeding during pregnancy (yes vs no)	5.0 (4.4 to 5.6)	4.7 (4.5 to 4.9)	0.3 (−0.4 to 1.0)	1.07 (0.93 to 1.23)
Placental abruption (yes vs no)	0.6 (0.4 to 0.8)	0.6 (0.5 to 0.7)	0.0 (−0.2 to 0.2)	1.00 (0.66 to 1.50)
Prelabor rupture of membranes (yes vs no)	2.4 (2.0 to 2.9)	2.5 (2.3 to 2.7)	0.0 (−0.5 to 0.5)	0.98 (0.80 to 1.21)
Mode of delivery (vs unassisted vaginal)				
Instrumental vaginal	12.9 (11.8 to 14.0)	12.3 (11.8 to 12.7)	0.6 (−0.6 to 1.9)	1.06 (0.95 to 1.19)
Planned cesarean	15.0 (13.8 to 16.1)	14.1 (13.7 to 14.6)	0.8 (−0.4 to 2.1)	1.07 (0.97 to 1.19)
Emergency cesarean	19.4 (18.1 to 20.7)	18.6 (18.2 to 19.1)	0.8 (−0.6 to 2.1)	1.05 (0.96 to 1.15)
Induced delivery (yes vs no)	19.5 (18.3 to 20.6)	19.0 (18.6 to 19.5)	0.4 (−0.8 to 1.7)	1.03 (0.95 to 1.12)
Infant death within 0-27 d (yes vs no)	0.3 (0.1 to 0.5)	0.1 (0.1 to 0.2)	0.2 (0.0 to 0.4)	2.67 (1.28 to 5.55)
Gestational age (vs term)				
Moderately preterm (32-36 wk)	5.2 (4.6 to 5.9)	5.8 (5.5 to 6.1)	−0.6 (−1.3 to 0.2)	0.90 (0.78 to 1.04)
Very preterm (<32 wk)	1.4 (1.1 to 1.8)	1.2 (1.1 to 1.4)	0.2 (−0.2 to 0.6)	1.15 (0.87 to 1.53)
Birth weight (<2500 g vs ≥2500 g)	4.7 (4.1 to 5.3)	4.8 (4.6 to 5.1)	−0.1 (−0.8 to 0.6)	0.98 (0.84 to 1.14)
BWGA percentile (vs 25.0-74.9 percentile)				
<10.0	21.8 (20.3 to 23.4)	20.7 (20.1 to 21.2)	1.2 (−0.5 to 2.9)	1.08 (0.97 to 1.19)
10.0-24.9	26.8 (25.2 to 28.4)	25.5 (24.9 to 26.1)	1.3 (−0.5 to 3.0)	1.07 (0.98 to 1.17)
75.0-89.9	18.3 (16.9 to 19.8)	19.8 (19.3 to 20.4)	−1.5 (−3.1 to 0.1)	0.91 (0.82 to 1.01)
≥90.0	11.8 (10.5 to 13.0)	14.2 (13.7 to 14.7)	−2.4 (−3.8 to −1.1)	0.80 (0.71 to 0.91)
Apgar score (<7 vs ≥7)	1.6 (1.2 to 1.9)	1.6 (1.4 to 1.7)	0.0 (−0.4 to 0.4)	1.00 (0.77 to 1.31)
Congenital malformation (vs none)				
Any	4.5 (3.9 to 5.1)	4.0 (3.8 to 4.2)	0.5 (−0.2 to 1.1)	1.13 (0.96 to 1.32)
Major	2.8 (2.3 to 3.3)	2.3 (2.1 to 2.5)	0.5 (−0.1 to 1.0)	1.20 (0.98 to 1.47)
Minor	1.8 (1.4 to 2.2)	1.8 (1.6 to 1.9)	0.0 (−0.4 to 0.5)	1.02 (0.80 to 1.31)
Sex of baby (boy vs girl)	49.7 (48.3 to 51.2)	51.7 (51.1 to 52.2)	−1.9 (−3.6 to −0.3)	0.93 (0.87 to 0.99)

Abbreviations: AAR, adjusted absolute risk; ARD, absolute risk difference; BWGA, birth weight for gestational age; OR, odds ratio; RRR, relative risk ratio.

^a^ Odds ratios for binary outcomes and RRRs for categorical outcomes, adjusted for maternal age at delivery, birth order, delivery year, maternal country of birth, previous spontaneous abortion, previous extrauterine pregnancy, and total number of stimulations.

### Exclusion of Double-Embryo Implantations

In an attempt to rule out a possible association of double implantations resulting in a singleton birth (vanishing twin syndrome), we repeated the analyses after excluding all multiple gestation pregnancies, which accounted for 8.0% of singleton births after DET and 0.3% of singleton births after SET in the cohort. In these analyses, the risk of major congenital malformations was significantly higher following DET vs SET (RRR, 1.24; 95% CI, 1.01-1.52), but the ARD of 0.5 percentage points was not statistically significant (95% CI, 0.0-1.1 percentage points) (Table 3). Children born after DET were less likely to be in the upper quartile of BWGA (percentile 75.0-89.9: RRR, 0.88 [95% CI, 0.78-0.98]; percentile ≥90.0: RRR, 0.82 [95% CI, 0.72-0.93]). The corresponding ARD was −2.0 percentage points [95% CI, −3.6 to −0.4 percentage points] for percentile 75.0-89.9 and −2.2 percentage points [95% CI, −3.6 to −0.8 percentage points] for percentile ≥90.0.

**Table 3.** Pregnancy and Birth Outcomes in Double-Embryo Transfer (DET) vs Single-Embryo Transfer (SET) Pregnancies, Excluding Possible Twin Pregnancies

	AAR, % (95% CI)		ARD, percentage points (95% CI)	RRR or OR (95% CI)^a^
	DET	SET		
Gestational hypertension (yes vs no)	5.9 (5.2 to 6.6)	5.8 (5.5 to 6.0)	0.1 (−0.6 to 0.9)	1.02 (0.89 to 1.18)
Preeclampsia (yes vs no)	3.8 (3.2 to 4.4)	3.9 (3.7 to 4.2)	−0.1 (−0.7 to 0.5)	0.97 (0.82 to 1.15)
Gestational diabetes (yes vs no)	1.9 (1.5 to 2.3)	2.1 (2.0 to 2.3)	−0.2 (−0.7 to 0.2)	0.90 (0.71 to 1.12)
Bleeding during pregnancy (yes vs no)	4.8 (4.2 to 5.4)	4.7 (4.4 to 4.9)	0.1 (−0.5 to 0.8)	1.03 (0.89 to 1.19)
Placental abruption (yes vs no)	0.6 (0.4 to 0.8)	0.6 (0.5 to 0.7)	0.0 (−0.2 to 0.3)	1.04 (0.69 to 1.59)
Prelabor rupture of membranes (yes vs no)	2.3 (1.9 to 2.8)	2.5 (2.3 to 2.7)	−0.1 (−0.6 to 0.4)	0.94 (0.76 to 1.17)
Mode of delivery (vs unassisted vaginal)				
Instrumental vaginal	12.6 (11.5 to 13.8)	12.3 (11.8 to 12.7)	0.4 (−0.9 to 1.6)	1.04 (0.92 to 1.17)
Planned cesarean	14.7 (13.6 to 15.9)	14.1 (13.6 to 14.5)	0.6 (−0.6 to 1.9)	1.06 (0.95 to 1.17)
Emergency cesarean	19.1 (17.8 to 20.4)	18.6 (18.1 to 19.1)	0.5 (−0.9 to 1.9)	1.03 (0.94 to 1.14)
Induced delivery (yes vs no)	19.5 (18.3 to 20.7)	19.0 (18.6 to 19.5)	0.4 (−0.9 to 1.8)	1.03 (0.95 to 1.12)
Infant death within 0-27 d (yes vs no)	0.3 (0.1 to 0.5)	0.1 (0.1 to 0.2)	0.2 (0.0 to 0.4)	2.68 (1.26 to 5.70)
Gestational age (vs term)				
Moderately preterm (32-36 wk)	5.2 (4.5 to 5.9)	5.8 (5.5 to 6.1)	−0.6 (−1.4 to 0.1)	0.89 (0.77 to 1.03)
Very preterm (<32 wk)	1.4 (1.0 to 1.8)	1.2 (1.1 to 1.4)	0.2 (−0.2 to 0.6)	1.14 (0.85 to 1.53)
Low birth weight (<2500 g vs ≥2500 g)	4.6 (4.0 to 5.3)	4.8 (4.6 to 5.1)	−0.2 (−0.9 to 0.5)	0.96 (0.82 to 1.12)
BWGA percentile (vs 25.0-74.9 percentile)				
<10.0	21.6 (20.0 to 23.2)	20.6 (20.1 to 21.2)	0.9 (−0.8 to 2.7)	1.06 (0.95 to 1.18)
10.0-24.9	26.6 (25.0 to 28.3)	25.5 (24.9 to 26.1)	1.1 (−0.7 to 2.9)	1.06 (0.96 to 1.16)
75.0-89.9	17.8 (16.3 to 19.3)	19.8 (19.3 to 20.4)	−2.0 (−3.6 to −0.4)	0.88 (0.78 to 0.98)
≥90.0	12.0 (10.7 to 13.2)	14.2 (13.7 to 14.7)	−2.2 (−3.6 to −0.8)	0.82 (0.72 to 0.93)
Apgar score (<7 vs ≥7)	1.6 (1.2 to 2.0)	1.6 (1.4 to 1.7)	0.1 (−0.4 to 0.5)	1.05 (0.80 to 1.37)
Congenital malformation (vs none)				
Any	4.6 (4.0 to 5.3)	4.0 (3.8 to 4.2)	0.6 (−0.1 to 1.3)	1.16 (0.99 to 1.37)
Major	2.8 (2.3 to 3.4)	2.3 (2.1 to 2.5)	0.5 (0.0 to 1.1)	1.24 (1.01 to 1.52)
Minor	1.9 (1.5 to 2.3)	1.8 (1.6 to 1.9)	0.1 (−0.3 to 0.6)	1.07 (0.83 to 1.37)
Sex of baby (boy vs girl)	49.7 (48.2 to 51.3)	51.7 (51.1 to 52.3)	−2.0 (−3.7 to −0.3)	0.92 (0.86 to 0.99)

Abbreviations: AAR, adjusted absolute risk; ARD, absolute risk difference; BWGA, birth weight for gestational age; OR, odds ratio; RRR, relative risk ratio.

^a^ Odds ratios for binary outcomes and RRRs for categorical outcomes, adjusted for maternal age at delivery, birth order, delivery year, maternal country of birth, previous spontaneous abortion, previous extrauterine pregnancy, and total number of stimulations.

### Comparison of ART Singletons (SET and DET) With Naturally Conceived Singletons

When comparing outcomes of both SET and DET pregnancies resulting in singleton birth with naturally conceived singletons (eTable 2 in the Supplement), ART was associated with significantly increased risks of bleeding during pregnancy, placental abruption, planned and emergency cesarean delivery, induced delivery, preterm birth, and low birth weight. The risk of prelabor rupture of membranes was higher after SET (OR, 1.25 [95% CI, 1.16-1.35]; ARD, 0.4 percentage points [95% CI, 0.2-0.5 percentage points]) but not DET (OR, 1.15 [95% CI, 0.96-1.38]; ARD, 0.2 percentage points [95% CI, −0.1 to 0.6 percentage points]). A higher risk of neonatal death was observed only after DET (OR, 2.02; 95% CI, 1.16-3.53), but the ARD of 0.1 percentage points was not statistically significant (95% CI, 0.0-0.3 percentage points). A small but significantly higher risk of low and high BWGA was observed in SET vs naturally conceived singletons. The risk of congenital malformations was higher after both DET (OR, 1.30 [95% CI, 1.14-1.48]; ARD 0.9 percentage points [95% CI, 0.4-1.5 percentage points]) and SET (OR, 1.15 [95% CI, 1.09-1.22]; ARD, 0.5 percentage points [95% CI, 0.3-0.7 percentage points]).

### Risk Assessment for SET vs DET Stratified by Fresh and Frozen Embryo Transfers

When the cohort was stratified on fresh and frozen cycles (Table 4), the risk of low birth weight was lower in fresh DET vs fresh SET (OR, 0.82 [95% CI, 0.69-0.97]; ARD, −1.0 percentage points [95% CI, −1.7 to −0.2 percentage points]) but higher in frozen DET vs frozen SET (OR, 1.64 [95% CI, 1.19-2.25]; ARD, 2.0 percentage points [95% CI, 0.5-3.5 percentage points]). Birth weight for gestational age was not significantly different between SET and DET in either fresh or frozen cycles. Further adjustment for endometrial preparation and years since embryo cryopreservation did not substantially alter the results for frozen cycles (eTable 3 in the Supplement).

**Table 4.** Pregnancy Outcomes in Double-Embryo Transfer (DET) vs Single-Embryo Transfer (SET) Pregnancies, Stratified by Fresh or Frozen Cycle and Cleavage Stage or Blastocyst Transfers

	Fresh cycles (n = 23 982)				Frozen cycles (n = 11 854)				*P* value^b^	Cleavage stage (n = 24 339)				Blastocyst transfers (n = 24 339)				P value^b^
	AAR, % (95% CI)		ARD, percentage points (95% CI)	RRR or OR (95% CI)^a^	AAR, % (95% CI)		ARD, percentage points (95% CI)	RRR or OR (95% CI)^a^		AAR, % (95% CI)		ARD, percentage points (95% CI)	RRR or OR (95% CI)^a^	AAR, % (95% CI)		ARD, percentage points (95% CI)	RRR or OR (95% CI)^a^	
	DET	SET			DET	SET				DET	SET			DET	SET			
Gestational age (vs term)									.09									.01
Moderately preterm	5.4 (4.6 to 6.1)	6.3 (6.0 to 6.7)	−1.0 (−1.8 to −0.1)	0.84 (0.72 to 0.98)	4.8 (3.4 to 6.2)	4.8 (4.4 to 5.2)	0.0 (−1.5 to 1.5)	1.00 (0.72 to 1.38)		5.2 (4.5 to 5.8)	5.8 (5.5 to 6.1)	−0.6 (−1.4 to 0.1)	0.89 (0.76 to 1.03)	6.0 (3.9 to 8.1)	5.8 (5.3 to 6.2)	0.3 (−1.9 to 2.4)	1.05 (0.72 to 1.54)	
Very preterm	1.3 (0.9 to 1.7)	1.3 (1.2 to 1.5)	0.0 (−0.4 to 0.4)	0.98 (0.71 to 1.35)	2.0 (1.0 to 2.9)	1.0 (0.8 to 1.2)	0.9 (−0.1 to 1.9)	1.90 (1.11 to 3.27)		1.3 (0.9 to 1.6)	1.3 (1.1 to 1.4)	0.0 (−0.4 to 0.4)	0.98 (0.72 to 1.35)	3.0 (1.5 to 4.5)	1.2 (0.9 to 1.4)	1.8 (0.3 to 3.4)	2.64 (1.50 to 4.63)	
Low birth weight (<2500 g vs ≥2500 g)	4.6 (3.9 to 5.3)	5.6 (5.3 to 5.9)	−1.0 (−1.7 to −0.2)	0.82 (0.69 to 0.97)	5.4 (3.9 to 6.9)	3.3 (3.0 to 3.7)	2.0 (0.5 to 3.5)	1.64 (1.19 to 2.25)	<.001	4.5 (3.8 to 5.1)	5.2 (4.8 to 5.5)	−0.7 (−1.4 to 0.0)	0.86 (0.73 to 1.01)	7.4 (5.1 to 9.7)	4.2 (3.8 to 4.6)	3.2 (0.9 to 5.5)	1.83 (1.29 to 2.60)	<.001
BWGA percentile (vs 25.0-74.9)									.45									.58
<10.0	22.6 (20.8 to 24.3)	22.7 (22.0 to 23.4)	−0.1 (−2.0 to 1.8)	0.99 (0.89 to 1.11)	18.8 (15.4 to 22.2)	16.3 (15.4 to 17.3)	2.5 (−1.0 to 6.0)	1.19 (0.94 to 1.50)		21.9 (20.3 to 23.6)	21.8 (21.1 to 22.5)	0.1 (−1.7 to 1.9)	1.01 (0.90 to 1.12)	20.7 (16.1 to 25.3)	18.5 (17.5 to 19.4)	2.3 (−2.5 to 7.0)	1.16 (0.86 to 1.55)	
10.0-24.9	27.9 (26.1 to 29.7)	27.0 (26.3 to 27.8)	0.9 (−1.1 to 2.8)	1.04 (0.95 to 1.15)	22.3 (18.9 to 25.8)	22.5 (21.5 to 23.5)	−0.2 (−3.8 to 3.4)	0.99 (0.80 to 1.22)		27.3 (25.6 to 29.0)	25.9 (25.1 to 26.7)	1.4 (−0.5 to 3.2)	1.07 (0.97 to 1.18)	23.5 (18.8 to 28.3)	24.9 (23.8 to 25.9)	−1.3 (−6.2 to 3.6)	0.93 (0.71 to 1.22)	
75.0-89.9	17.5 (15.9 to 19.1)	17.8 (17.1 to 18.6)	−0.4 (−2.1 to 1.4)	0.98 (0.86 to 1.10)	21.0 (17.7 to 24.2)	23.1 (22.1 to 24.1)	−2.1 (−5.5 to 1.3)	0.88 (0.72 to 1.08)		17.8 (16.3 to 19.3)	18.8 (18.1 to 19.5)	−0.9 (−2.6 to 0.7)	0.94 (0.84 to 1.05)	22.1 (17.5 to 26.7)	21.6 (20.6 to 22.6)	0.5 (−4.3 to 5.2)	1.03 (0.78 to 1.36)	
≥90.0	10.8 (9.5 to 12.1)	11.8 (11.1 to 12.4)	−1.0 (−2.5 to 0.5)	0.90 (0.78 to 1.05)	15.1 (12.2 to 18.0)	18.1 (17.1 to 19.0)	−3.0 (−6.0 to 0.0)	0.80 (0.63 to 1.02)		11.4 (10.1 to 12.6)	13.2 (12.6 to 13.8)	−1.8 (−3.3 to −0.4)	0.84 (0.73 to 0.97)	14.6 (10.5 to 18.6)	15.9 (15.0 to 16.9)	−1.4 (−5.5 to 2.8)	0.90 (0.64 to 1.26)	

Abbreviations: AAR, adjusted absolute risk; ARD, absolute risk difference; BWGA, birth weight for gestational age; OR, odds ratio; RRR, relative risk ratio.

^a^ Odds ratios for binary outcomes and RRRs for categorical outcomes.

^b^ *P* value for interaction. Likelihood ratio tests for interaction between each outcome and fresh or frozen cycle with cleavage stage embryos, or fresh or frozen cycle with blastocyst embryos. Models were adjusted for maternal age at delivery, birth order, delivery year, maternal country of birth, previous spontaneous abortion, previous extrauterine pregnancy, and total number of stimulations.

### Risk Assessment for SET vs DET Stratified by Cleavage Stage or Blastocyst Transfers

Within cycles at cleavage stage, DET was not significantly associated with preterm birth, low birth weight, or BWGA (Table 4). Among births following transfer of embryos at the blastocyst stage, DET was associated with a higher risk of low birth weight (OR, 1.83 [95% CI, 1.29-2.60]; ARD, 3.2 percentage points [95% CI, 0.9-5.5 percentage points]) and very preterm birth (RRR, 2.64 [95% CI, 1.50-4.63]; ARD 1.8 percentage points [95% CI, 0.3-3.4 percentage points]). No significant differences in BWGA were observed between DET and SET using blastocysts (Table 4).

### Risk Assessment for SET and DET in Double Stratification by Fresh or Frozen and Cleavage Stage or Blastocyst Transfers

Among fresh cleavage stage cycles (eTable 4 in the Supplement), the risk of low birth weight was lower in DET vs SET (OR, 0.76 [95% CI, 0.63-0.91]; ARD, −1.3 percentage points [95% CI, −2.1 to −0.5 percentage points). There were no statistically significant differences in preterm birth or BWGA. There were no observed differences in outcomes between SET and DET in transfers of frozen embryos at the cleavage stage, whereas DET using frozen blastocysts was associated with a higher risk of low birth weight (OR, 2.92 [95% CI, 1.67-5.11]; ARD, 5.3 percentage points [95% CI, 1.2-9.5 percentage points]) but not low BWGA (eTable 4 in the Supplement).

### Sensitivity Analyses

When restricting to first births only (eTable 5 in the Supplement), children born after DET were more likely to be in the lower quartile of BWGA (percentile <10.0: RRR, 1.13 [95% CI, 1.00-1.27]; ARD, 2.3 percentage points [95% CI, 0.0-4.5 percentage points]; percentile 10.0-24.9: RRR, 1.14 [95% CI, 1.02-1.27]; ARD, 2.7 percentage points [95% CI, 0.4-5.0 percentage points]). The remaining comparisons had similar results to those shown in Table 2. Further adjustment for fertilization method, maternal education, BMI, and smoking during pregnancy using multiple imputation did not substantially alter the main results (eTable 6 in the Supplement).

## Discussion

The results of this cohort study show a higher incidence of adverse outcomes in singletons born through ART compared with natural conception.^20^ However, with this study, we also explored whether a DET had an adverse association with outcomes in singleton births after IVF and ICSI treatments, focusing on neonatal and perinatal risk factors. Both absolute risks and relative risks (ORs or RRRs of adverse outcomes were calculated on the basis of the transfer of either 1 or 2 embryos at different developmental stages depending on the length of in vitro embryo culture and according to the use of either fresh or frozen and thawed embryos. In this population-based study cohort, the ARTs were performed according to the Swedish National Board of Health and Welfare’s policy on SET established in 2003, wherein, as a general recommendation, patients undergoing ART should receive SET and only patients with a low risk for twin pregnancy would be candidates for DET.^21^ Adherence to policy is clearly reflected in our cohort characteristics, where the number of DETs are low and have been decreasing over time. Double-embryo transfer singleton births were more common in older patients, who also received more ICSI treatments. The transfer of blastocysts was 3.8 times and frozen cycles 2.0 times more frequent in SET. The group receiving SET had a more favorable fertility prognosis, and presumably, although our model was adjusted for age and several other potential confounders, a lower embryo quality in the DET group could explain the observed negative outcomes. Results could also be affected by the study design, demographic characteristics, study size, and unmeasured confounding. Our cohort is taken from nationwide registers of close to 100% of all ART treatments and births in Sweden; thus, our findings on outcome incidence are based on comprehensive data.

It has been previously reported that singleton pregnancies resulting from DET may have a significant negative association with birth weight and preterm birth compared with SET.^22^ Furthermore, previous research suggested that an increase in the number of embryos transferred will increase the risk for growth restriction in singletons.^23^ A recent study showed improved perinatal outcomes after SET compared with DET and compared with multiple-embryo transfers; more specifically, the risk of preterm birth and low birth weight decreased when a lower number of embryos were transferred.^24^

In our cohort of singleton children born after DET, we observed higher relative risks for adverse outcomes associated with the health of the newborn, such as neonatal death and major congenital malformations, compared with SET. However, the absolute risks of these outcomes were low, and the ARDs were small and not significant. Although we could observe a slight tendency toward growth restriction when IVF and ICSI were compared with spontaneous conception, we found no differences in BWGA between DET and SET in the full data set. In this cohort, as in previous reports, we observed a tendency toward higher BWGA when frozen cycles were compared with fresh cycles.^25^

With regard to blastocyst transfers, a recent meta-analysis indicated that the general risk of both low birth weight and preterm birth in singleton pregnancies, independent of the number of embryos transferred, was lower after cryopreserved blastocyst transfers compared with fresh blastocyst transfers.^28^ Furthermore, the transfer of frozen and thawed blastocysts showed a significantly higher rate of neonates large for gestational age and a significantly lower rate of neonates small for gestational age.^28^ In our study, DET was not significantly associated with BWGA compared with SET, but we observed a higher relative risk of very preterm birth associated with DET with fresh blastocysts and an increased risk of both low birth weight and preterm birth after DET of frozen and thawed blastocysts. A recent meta-analysis in the United Kingdom reported that singletons born after frozen embryo transfer are more likely to be large for gestational age than singletons born after fresh transfer.^29^ The higher incidence of low birth weight and very preterm birth observed after DET could be due to parental infertility-related factors affecting embryo quality or perhaps the impact of prolonged culture alone or in combination with cryopreservation and blastocyst transfer.

A slightly decreased risk of low birth weight was observed in DET fresh cleavage stage embryo cycles vs SET, which was not observed after blastocyst replacements. In contrast, the absolute risk of low birth weight and preterm birth after frozen SET cycles at both cleavage and blastocyst stages was similar to that after natural conception. The differences between fresh and frozen cycles might be interpreted as a direct association with cryopreservation,^30^ although various strategies during stimulation, endometrial preparation, and embryo selection could also be influencing the outcome.

Vanishing twin syndrome takes place after DET when both transferred embryos implant but only 1 of the embryos progresses during pregnancy. Vanishing twin syndrome has been shown to occur in more than 10% of IVF multiple gestations as confirmed by ultrasound^31^ and has been identified as a major cause of singleton low birth weight and preterm birth after transfer of multiple embryos.^28^ More research is needed to identify a mechanism to explain both the pregnancy loss and the influence on the surviving twin. However, excluding all known cases of double implantations (8% of singleton births after DET) from our cohort did not attenuate the observed risks of neonatal death, which suggests an adverse association of DET independent of vanishing twin syndrome.

### Limitations

This study has some limitations. First, a major limitation is the lack of information on embryo quality as previously described. Second, it was not possible to further investigate the associations by type of infertility because this information was only available for a subset of the cohort. Third, the study had limited power to detect differences in analysis stratified both by fresh or frozen and by cleavage stage or blastocyst transfer.

## Conclusions

Although few significant differences in perinatal and obstetric outcomes were observed in this nationwide, population-based cohort study of singletons resulting from DET vs SET, we found a higher risk of neonatal death after DET. In frozen cycles, DET was associated with a higher risk of low birth weight, and after blastocyst transfers, DET was associated with a risk of very preterm birth and low birth weight. The observed differences in risk between SET and DET were highest after frozen blastocyst transfer.

Although neonatal death is a serious detrimental outcome, the absolute risk after DET is still very low, especially in relation to the general risks of ART and twin pregnancies. We conclude that although the results support the use of SET, the observed higher risk after DET should not in itself deter from the use of DET in women who are older or otherwise have a low reproductive potential.

## Notes

Supplement. **eTable 1.** *International Classification of Diseases, Tenth Revision* Codes for Major and Minor Malformations **eTable 2.** Pregnancy and Birth Outcomes in Double-Embryo Transfer (DET) or Single-Embryo Transfer (SET) vs Spontaneous Pregnancies **eTable 3.** Pregnancy and Birth Outcomes in Double-Embryo Transfer (DET) vs Single-Embryo Transfer (SET) Pregnancies Among Frozen Cycles Only **eTable 4.** Pregnancy Outcomes in Double-Embryo Transfer (DET) vs Single-Embryo Transfer (SET) Pregnancies Stratified by Fresh or Frozen Cycle and by Cleavage Stage or Blastocyst Transfer **eTable 5.** Pregnancy and Birth Outcomes in Double-Embryo Transfer (DET) vs Single-Embryo Transfer (SET) Pregnancies Among First Births Only **eTable 6.** Sensitivity Analysis of Table 2, Including Adjustments by Means of Multiple Imputation
