# Supplementary material for: Obstetric and Perinatal Outcomes of Singleton Births Following Single- vs Double-Embryo Transfer in Sweden
Source: JAMA Pediatr. 2022 Dec 5:e224787. Online ahead of print. doi: 10.1001/jamapediatrics.2022.4787 (PMC9857532; doi:10.1001/jamapediatrics.2022.4787)
Supplement: Supplement. — eTable 1. International Classification of Diseases, Tenth Revision Codes for Major and Minor Malformations eTable 2. Pregnancy and Birth Outcomes in Double-Embryo Transfer (DET) or Single-Embryo Transfer (SET) vs Spontaneous Pregnancies eTable 3. Pregnancy and Birth Outcomes in Double-Embryo Transfer (DET) vs Single-Embryo Transfer (SET) Pregnancies Among Frozen Cycles Only eTable 4. Pregnancy Outcomes in Double-Embryo Transfer (DET) vs Single-Embryo Transfer (SET) Pregnancies Stratified by Fresh or Frozen Cycle and by Cleavage Stage or Blastocyst Transfer eTable 5. Pregnancy and Birth Outcomes in Double-Embryo Transfer (DET) vs Single-Embryo Transfer (SET) Pregnancies Among First Births Only eTable 6. Sensitivity Analysis of Table 2, Including Adjustments by Means of Multiple Imputation [file jamapediatr-e224787-s001.pdf]

Supplemental Online Content

Rodriguez-Wallberg KA, Palomares AR, Nilsson HP, et al. Obstetric and perinatal outcomes of singleton births following single- vs double-embryo transfer in Sweden. *JAMA Pediatr*. Published online December 5, 2022. doi:10.1001/jamapediatrics.2022.4787

eTable 1. *International Classification of Diseases, Tenth Revision* Codes for Major and Minor Malformations

eTable 2. Pregnancy and Birth Outcomes in Double-Embryo Transfer (DET) or Single-Embryo Transfer (SET) vs Spontaneous Pregnancies

eTable 3. Pregnancy and Birth Outcomes in Double-Embryo Transfer (DET) vs Single-Embryo Transfer (SET) Pregnancies Among Frozen Cycles Only

eTable 4. Pregnancy Outcomes in Double-Embryo Transfer (DET) vs Single-Embryo Transfer (SET) Pregnancies Stratified by Fresh or Frozen Cycle and by Cleavage Stage or Blastocyst Transfer

eTable 5. Pregnancy and Birth Outcomes in Double-Embryo Transfer (DET) vs Single-Embryo Transfer (SET) Pregnancies Among First Births Only

eTable 6. Sensitivity Analysis of Table 2, Including Adjustments by Means of Multiple Imputation

This supplemental material has been provided by the authors to give readers additional information about their work.

© 2022 Rodriguez-Wallberg KA, et al. *JAMA Pediatrics.*

eTable 1. *International Classification of Diseases, Tenth Revision* Codes for Major and Minor Malformations

Organ System	Major malformations	Minor malformations
Nervous System	Q00-Q07	-
Eye	Q100, Q104, Q106-Q109, Q11-Q12, Q130-Q134, Q136-Q139, Q14-Q15	Q101-Q103, Q105, Q135
Ear, Face and Neck	Q16, Q176-Q178, Q183, Q188	Q170-Q175, Q179, Q180-Q182, Q184-Q187, Q189
Congenital Heart Defects	Q20-Q23, Q240-Q245, Q247-Q249, Q25, Q260, Q262-Q269	Q250 and Q256 if GA <37w, Q246, Q261
Respiratory System	Q30, Q310-Q313, Q315-Q319, Q321, Q323-Q329, Q330, Q332-Q339, Q34	Q314, Q320, Q322, Q331
Orofacial Clefts	Q35-Q37	-
Digestive System	Q380, Q383-Q389, Q39, Q402-Q409, Q41-Q42, Q431-Q439, Q440-Q443, Q445-Q449, Q45, Q790	Q381-Q382, Q385A, Q400-Q401, Q430, Q444
Abdominal Wall Defects	Q792-Q793, Q795	-
Urinary System	Q60, Q611-Q619, Q620-Q626, Q628-Q629, Q630-Q632, Q634-Q639, Q64, Q794	Q610, Q627, Q633
Genital Organs	Q500, Q503-Q504, Q506-Q509, Q51, Q520-Q522, Q524, Q526, Q528-Q529, Q540-Q543, Q545-Q549, Q55-Q56	Q501-Q502, Q505, Q523, Q525, Q527, Q53, Q544
Limb	Q650-Q652, Q657, Q660, Q679, Q681-Q682, Q686-Q689, Q70-Q74	Q653-Q656, Q658-Q659, Q661-Q669, Q670-Q678, Q680, Q683-Q685
Other musculoskeletal	Q750-Q751, Q754-Q759, Q761-Q764, Q766-Q769, Q77, Q78, Q791, Q792-Q793, Q795-Q799	Q752-Q753, Q760, Q765, Q791, Q794
Other	Q271-Q279, Q28, Q80-Q81, Q820-Q824, Q826-Q829, Q83-Q85, Q860, Q890, Q893-Q898	Q270, Q825, Q891-Q892, Q899
Teratogenic/genetic syndromes, microdeletions and chromosomal abnormalities (excluded)	Q861-Q869, Q87, Q90-Q99, P350-P352, P371, D821	-

Definitions based on the European network of population-based registries for the epidemiological surveillance of congenital anomalies (EUROCAT) guide 1.4 version 23.09.2020 (https://eu-rd-platform.jrc.ec.europa.eu/eurocat/data-collection/guidelines-for-data-registration_en)

© 2022 Rodriguez-Wallberg KA, et al. *JAMA Pediatrics.*

eTable 2. Pregnancy and Birth Outcomes in Double-Embryo Transfer (DET) or Single-Embryo Transfer (SET) vs Spontaneous Pregnancies

	AAR, % (95% CI)			ARD, percentage points (95% CI)		RRR or OR (95% CI)[a]	
	DET	SET	Spont.	DET vs spont.	SET vs spont.	DET vs spont.	SET vs spont.
Gestational hypertension (yes vs no)	3.9 (3.5 to 4.4)	4.1 (3.9 to 4.3)	4.1 (4.1 to 4.1)	-0.2 (-0.6 to 0.3)	0.0 (-0.2 to 0.2)	0.95 (0.85 to 1.07)	1.01 (0.96 to 1.06)
Preeclampsia (yes vs no)	2.7 (2.3 to 3.0)	2.9 (2.7 to 3.0)	2.8 (2.7 to 2.8)	-0.1 (-0.5 to 0.3)	0.1 (-0.1 to 0.3)	0.96 (0.83 to 1.10)	1.04 (0.98 to 1.10)
Gestational diabetes (yes vs no)	1.8 (1.5 to 2.1)	1.9 (1.8 to 2.1)	1.8 (1.8 to 1.9)	-0.1 (-0.4 to 0.3)	0.1 (-0.1 to 0.2)	0.97 (0.80 to 1.16)	1.04 (0.96 to 1.13)
Bleeding during pregnancy (yes vs no)	3.7 (3.3 to 4.1)	3.4 (3.2 to 3.6)	2.0 (2.0 to 2.1)	1.6 (1.2 to 2.0)	1.4 (1.2 to 1.6)	1.83 (1.63 to 2.06)	1.70 (1.60 to 1.80)
Placental abruption (yes vs no)	0.6 (0.4 to 0.8)	0.6 (0.5 to 0.7)	0.3 (0.3 to 0.3)	0.3 (0.1 to 0.5)	0.3 (0.2 to 0.3)	1.83 (1.29 to 2.59)	1.82 (1.56 to 2.12)
Prelabor rupture of membranes (yes vs no)	1.8 (1.5 to 2.1)	2.0 (1.8 to 2.1)	1.6 (1.6 to 1.6)	0.2 (-0.1 to 0.6)	0.4 (0.2 to 0.5)	1.15 (0.96 to 1.38)	1.25 (1.16 to 1.35)
Mode of delivery (vs unassisted vaginal)							
Instrumental vaginal	7.6 (6.9 to 8.2)	7.6 (7.3 to 7.8)	7.9 (7.8 to 7.9)	-0.3 (-1.0 to 0.3)	-0.3 (-0.6 to 0.0)	0.95 (0.86 to 1.05)	0.95 (0.91 to 0.99)
Planned cesarean	12.1 (11.2 to 13.0)	11.3 (10.9 to 11.7)	8.5 (8.5 to 8.6)	3.6 (2.7 to 4.4)	2.8 (2.4 to 3.2)	1.49 (1.36 to 1.62)	1.38 (1.33 to 1.43)
Emergency cesarean	12.5 (11.6 to 13.3)	12.2 (11.8 to 12.5)	10.9 (10.8 to 11.0)	1.5 (0.7 to 2.4)	1.3 (0.9 to 1.6)	1.17 (1.08 to 1.26)	1.14 (1.10 to 1.18)
Induced delivery (yes vs no)	17.3 (16.3 to 18.3)	16.7 (16.3 to 17.1)	14.3 (14.2 to 14.4)	3.0 (2.0 to 4.0)	2.4 (2.0 to 2.8)	1.25 (1.17 to 1.34)	1.20 (1.16 to 1.24)
Infant death within 0-27 days (yes vs no)	0.2 (0.1 to 0.4)	0.1 (0.1 to 0.2)	0.1 (0.1 to 0.1)	0.1 (0.0 to 0.3)	0.0 (0.0 to 0.0)	2.02 (1.16 to 3.53)	1.01 (0.72 to 1.42)
Gestational age (vs term)							
Moderately preterm (32-36 weeks)	4.5 (4.0 to 5.0)	5.3 (5.0 to 5.5)	3.9 (3.9 to 4.0)	0.6 (0.0 to 1.1)	1.3 (1.1 to 1.6)	1.15 (1.02 to 1.31)	1.36 (1.30 to 1.43)
Very preterm (<32 weeks)	1.1 (0.9 to 1.4)	1.1 (0.9 to 1.2)	0.7 (0.6 to 0.7)	0.5 (0.2 to 0.7)	0.4 (0.3 to 0.5)	1.72 (1.36 to 2.18)	1.59 (1.43 to 1.78)
Low birth weight (<2500g vs ≥2500g)	3.8 (3.3 to 4.2)	4.1 (3.9 to 4.3)	3.0 (3.0 to 3.0)	0.8 (0.3 to 1.2)	1.1 (0.9 to 1.3)	1.27 (1.12 to 1.44)	1.38 (1.30 to 1.46)
BWGA percentile (vs 25.0-74.9 percentile)							
<10.0	18.8 (17.6 to 20.1)	18.7 (18.2 to 19.3)	17.8 (17.7 to 17.8)	1.1 (-0.2 to 2.4)	1.0 (0.4 to 1.5)	1.08 (0.99 to 1.18)	1.07 (1.03 to 1.11)
10.0-24.9	25.3 (23.9 to 26.7)	24.5 (23.9 to 25.1)	24.4 (24.3 to 24.5)	0.9 (-0.5 to 2.3)	0.1 (-0.5 to 0.7)	1.05 (0.97 to 1.13)	1.00 (0.97 to 1.04)
75.0-89.9	19.3 (17.9 to 20.8)	21.1 (20.5 to 21.7)	20.2 (20.2 to 20.3)	-0.9 (-2.3 to 0.5)	0.8 (0.2 to 1.4)	0.94 (0.86 to 1.03)	1.05 (1.01 to 1.09)
≥90.0	13.3 (12.0 to 14.6)	15.7 (15.1 to 16.2)	14.3 (14.2 to 14.3)	-1.0 (-2.3 to 0.3)	1.4 (0.8 to 2.0)	0.92 (0.82 to 1.03)	1.12 (1.07 to 1.17)
Apgar score (<7 vs ≥7)	1.2 (0.9 to 1.4)	1.3 (1.2 to 1.4)	1.2 (1.2 to 1.2)	0.0 (-0.3 to 0.2)	0.1 (0.0 to 0.2)	0.97 (0.77 to 1.22)	1.07 (0.98 to 1.18)
Congenital malformation (vs none)							
Any	4.2 (3.6 to 4.7)	3.7 (3.5 to 3.9)	3.2 (3.2 to 3.3)	0.9 (0.4 to 1.5)	0.5 (0.3 to 0.7)	1.30 (1.14 to 1.48)	1.15 (1.09 to 1.22)
Major	2.7 (2.2 to 3.1)	2.2 (2.1 to 2.4)	1.9 (1.9 to 2.0)	0.8 (0.3 to 1.2)	0.3 (0.1 to 0.5)	1.40 (1.18 to 1.66)	1.16 (1.08 to 1.26)
Minor	1.6 (1.3 to 1.9)	1.5 (1.4 to 1.7)	1.4 (1.3 to 1.4)	0.2 (-0.1 to 0.5)	0.2 (0.0 to 0.3)	1.17 (0.95 to 1.43)	1.13 (1.04 to 1.24)
Sex of baby (boy vs girl)	50.2 (48.8 to 51.5)	51.6 (51.0 to 52.2)	51.4 (51.4 to 51.5)	-1.3 (-2.7 to 0.1)	0.2 (-0.4 to 0.7)	0.95 (0.90 to 1.00)	1.01 (0.98 to 1.03)

Abbreviations: AAR, adjusted absolute risk; ARD, adjusted risk difference; BWGA, birth weight for gestational age; OR, odds ratio; RRR, relative risk ratio.

[a]Odds ratios for binary outcomes and relative risk ratios for categorical outcomes, adjusted for maternal age at delivery, birth order, delivery year, maternal country of birth, previous spontaneous abortion and previous extra-uterine pregnancy.

© 2022 Rodriguez-Wallberg KA, et al. *JAMA Pediatrics.*

eTable 3. Pregnancy and Birth Outcomes in Double-Embryo Transfer (DET) vs Single-Embryo Transfer (SET) Pregnancies, Among Frozen Cycles Only

	Model 1				Model 2			
	AAR, % (95% CI)		ARD, percentage points (95% CI)	RRR or OR (95% CI)[a]	AAR, % (95% CI)		ARD, percentage points (95% CI)	RRR or OR (95% CI)[a]
	DET	SET			DET	SET		
Gestational age (vs term)								
Moderately preterm (32-36 weeks)	4.4 (3.1 to 5.8)	4.8 (4.4 to 5.2)	-0.4 (-1.8 to 1.1)	0.92 (0.66 to 1.28)	4.3 (3.0 to 5.6)	4.8 (4.4 to 5.2)	-0.5 (-1.9 to 0.9)	0.90 (0.64 to 1.25)
Very preterm (<32 weeks)	1.7 (0.8 to 2.6)	1.0 (0.8 to 1.2)	0.7 (-0.2 to 1.7)	1.75 (0.96 to 3.17)	1.7 (0.8 to 2.6)	1.0 (0.8 to 1.2)	0.7 (-0.3 to 1.6)	1.70 (0.93 to 3.09)
Low birth weight (<2500g vs ≥2500g)	4.9 (3.4 to 6.4)	3.2 (2.9 to 3.5)	1.7 (0.2 to 3.2)	1.57 (1.12 to 2.19)	4.7 (3.3 to 6.2)	3.2 (2.9 to 3.5)	1.6 (0.1 to 3.0)	1.51 (1.08 to 2.12)
BWGA percentile (vs 25.0-74.9 percentile)								
<10.0	18.4 (15.0 to 21.8)	15.4 (14.6 to 16.3)	3.0 (-0.6 to 6.5)	1.24 (0.97 to 1.58)	18.3 (14.9 to 21.7)	15.4 (14.5 to 16.3)	2.9 (-0.6 to 6.4)	1.24 (0.97 to 1.58)
10.0-24.9	21.8 (18.2 to 25.3)	22.1 (21.1 to 23.1)	-0.3 (-4.0 to 3.4)	0.98 (0.79 to 1.22)	21.8 (18.3 to 25.4)	22.1 (21.1 to 23.1)	-0.3 (-4.0 to 3.4)	0.98 (0.79 to 1.22)
75.0-89.9	22.0 (18.4 to 25.5)	23.6 (22.6 to 24.6)	-1.7 (-5.4 to 2.0)	0.91 (0.73 to 1.13)	22.0 (18.5 to 25.6)	23.6 (22.6 to 24.6)	-1.6 (-5.2 to 2.1)	0.92 (0.74 to 1.14)
≥90.0	15.6 (12.5 to 18.7)	18.6 (17.7 to 19.6)	-3.0 (-6.3 to 0.2)	0.81 (0.63 to 1.03)	15.5 (12.4 to 18.5)	18.7 (17.7 to 19.6)	-3.2 (-6.4 to 0.0)	0.80 (0.62 to 1.02)

Analyses restricted to frozen cycles with information on endometrial preparation and years since embryo cryopreservation, n=11 576.
Abbreviations: AAR, adjusted absolute risk; ARD, adjusted risk difference; BWGA, birth weight for gestational age; OR, odds ratio; RRR, relative risk ratio
[a]Odds ratios for binary outcomes and relative risk ratios for categorical outcomes. Model 1 adjusted for maternal age at delivery, birth order, delivery year, maternal country of birth, previous spontaneous abortion, previous extra-uterine pregnancy and total number of stimulations. Model 2 adjusted similarly to as in Model 1, with additional adjustments for endometrial preparation (artificial, natural cycle) and years since embryo cryopreservation (<1, 1-2, 3 or more).

© 2022 Rodriguez-Wallberg KA, et al. *JAMA Pediatrics.*

eTable 4. Pregnancy Outcomes in Double-Embryo Transfer (DET) vs Single-Embryo Transfer (SET) Pregnancies Stratified by Fresh or Frozen Cycle and by Cleavage Stage or Blastocyst Transfer

	Fresh cycles, cleavage stage, n=19 109				Frozen cycles, cleavage stage, n=5 230				*P* value for interaction[b]
	AAR, % (95% CI)		ARD, percentage points (95% CI)	RRR or OR (95% CI)[a]	AAR, % (95% CI)		ARD, percentage points (95% CI)	RRR or OR (95% CI)[a]	
	DET	SET			DET	SET			
Gestational age (vs term)									
Moderately preterm (32-36 weeks)	5.4 (4.7 to 6.2)	6.2 (5.8 to 6.6)	-0.7 (-1.7 to 0.2)	0.87 (0.73 to 1.04)	4.2 (2.7 to 5.7)	4.5 (3.9 to 5.1)	-0.3 (-1.9 to 1.3)	0.93 (0.62 to 1.39)	0.50
Very preterm (<32 weeks)	1.2 (0.8 to 1.5)	1.3 (1.1 to 1.5)	-0.2 (-0.6 to 0.3)	0.88 (0.61 to 1.26)	1.6 (0.6 to 2.6)	1.1 (0.8 to 1.5)	0.4 (-0.6 to 1.5)	1.40 (0.69 to 2.81)	
Low birth weight (<2500g vs ≥2500g)	4.3 (3.7 to 5.0)	5.6 (5.3 to 6.0)	-1.3 (-2.1 to -0.5)	0.76 (0.63 to 0.91)	4.7 (3.1 to 6.3)	3.7 (3.1 to 4.3)	1.0 (-0.7 to 2.7)	1.29 (0.87 to 1.91)	0.02
BWGA percentile (vs 25.0-74.9 percentile)									
<10.0	22.7 (20.8 to 24.5)	23.1 (22.3 to 24.0)	-0.4 (-2.5 to 1.7)	0.97 (0.86 to 1.10)	19.0 (15.2 to 22.9)	17.2 (15.7 to 18.7)	1.9 (-2.2 to 6.0)	1.14 (0.87 to 1.50)	0.73
10.0-24.9	27.8 (25.9 to 29.7)	27.3 (26.4 to 28.2)	0.5 (-1.6 to 2.7)	1.03 (0.92 to 1.14)	21.9 (18.0 to 25.7)	21.6 (20.0 to 23.1)	0.3 (-3.8 to 4.5)	1.02 (0.80 to 1.30)	
75.0-89.9	17.3 (15.6 to 19.0)	17.6 (16.8 to 18.4)	-0.3 (-2.2 to 1.6)	0.98 (0.86 to 1.12)	19.7 (16.1 to 23.3)	21.6 (20.1 to 23.2)	-1.9 (-5.8 to 1.9)	0.89 (0.70 to 1.13)	
≥90.0	10.5 (9.1 to 11.9)	11.6 (10.9 to 12.4)	-1.2 (-2.7 to 0.4)	0.89 (0.75 to 1.05)	14.2 (11.1 to 17.3)	17.2 (15.8 to 18.7)	-3.0 (-6.4 to 0.4)	0.79 (0.60 to 1.04)	
	Fresh cycles, blastocyst, n=4 848				Frozen cycles, blastocyst, n=6 543				*P* value for interaction[b]
	AAR, % (95% CI)		ARD, percentage points (95% CI)	RRR or OR (95% CI)[a]	AAR, % (95% CI)		ARD, percentage points (95% CI)	RRR or OR (95% CI)[a]	
	DET	SET			DET	SET			
Gestational age (vs term)									
Moderately preterm (32-36 weeks)	5.2 (2.7 to 7.7)	6.6 (5.9 to 7.3)	-1.4 (-4.0 to 1.2)	0.78 (0.47 to 1.30)	7.1 (3.3 to 10.8)	5.0 (4.5 to 5.6)	2.1 (-1.7 to 5.9)	1.44 (0.80 to 2.59)	0.21
Very preterm (<32 weeks)	3.2 (1.0 to 5.4)	1.4 (1.1 to 1.8)	1.7 (-0.5 to 4.0)	2.26 (1.06 to 4.79)	3.6 (0.7 to 6.5)	1.0 (0.7 to 1.2)	2.6 (-0.3 to 5.6)	3.83 (1.59 to 9.25)	
Low birth weight (<2500g vs ≥2500g)	7.6 (4.5 to 10.7)	5.6 (4.9 to 6.3)	2.0 (-1.2 to 5.2)	1.39 (0.87 to 2.21)	8.4 (4.3 to 12.5)	3.1 (2.6 to 3.5)	5.3 (1.2 to 9.5)	2.92 (1.67 to 5.11)	0.05
BWGA percentile (vs 25.0-74.9 percentile)									
<10.0	23.2 (17.1 to 29.2)	21.4 (19.9 to 22.9)	1.8 (-4.4 to 8.0)	1.11 (0.78 to 1.58)	15.6 (8.4 to 22.8)	15.7 (14.5 to 16.9)	-0.1 (-7.4 to 7.3)	0.99 (0.57 to 1.75)	0.79
10.0-24.9	24.5 (18.3 to 30.8)	26.8 (25.2 to 28.4)	-2.2 (-8.7 to 4.2)	0.89 (0.63 to 1.26)	25.9 (17.5 to 34.2)	22.9 (21.6 to 24.2)	2.9 (-5.5 to 11.4)	1.17 (0.75 to 1.83)	

© 2022 Rodriguez-Wallberg KA, et al. *JAMA Pediatrics.*

75.0-89.9	19.3 (13.7 to 24.9)	18.3 (16.9 to 19.8)	1.0 (-4.8 to 6.8)	1.07 (0.73 to 1.55)	25.3 (17.4 to 33.2)	24.3 (23.0 to 25.6)	1.0 (-7.0 to 9.1)	1.06 (0.69 to 1.62)
≥90.0	12.8 (8.0 to 17.7)	12.2 (11.0 to 13.5)	0.6 (-4.4 to 5.6)	1.06 (0.67 to 1.66)	16.7 (9.6 to 23.8)	18.7 (17.5 to 20.0)	-2.0 (-9.3 to 5.2)	0.87 (0.51 to 1.47)

Abbreviations: AAR, adjusted absolute risk; ARD, adjusted risk difference; BWGA, birth weight for gestational age;OR, odds ratio; RRR, relative risk ratio:

[a]Odds ratios for binary outcomes and relative risk ratios for categorical outcomes, adjusted for maternal age at delivery, birth order, delivery year, maternal country of birth, previous spontaneous abortion, previous extra-uterine pregnancy and total number of stimulations.

[b]Likelihood ratio tests for interaction between each outcome and fresh or frozen cycle with cleavage stage embryos, or fresh or frozen cycle with blastocyst embryos, respectively.

© 2022 Rodriguez-Wallberg KA, et al. *JAMA Pediatrics*.

eTable 5. Pregnancy and Birth Outcomes in Double-Embryo Transfer (DET) vs Single-Embryo Transfer (SET) pregnancies Among First Births Only

	AAR, % (95% CI)		ARD, percentage points (95% CI)	RRR or OR (95% CI)[a]
	DET	SET		
Gestational hypertension (yes vs no)	7.2 (6.2 to 8.1)	6.9 (6.5 to 7.2)	0.3 (-0.7 to 1.3)	1.04 (0.89 to 1.22)
Preeclampsia (yes vs no)	4.6 (3.9 to 5.4)	4.9 (4.6 to 5.2)	-0.2 (-1.1 to 0.6)	0.95 (0.79 to 1.14)
Gestational diabetes (yes vs no)	1.9 (1.5 to 2.4)	2.2 (2.0 to 2.4)	-0.3 (-0.8 to 0.2)	0.87 (0.66 to 1.13)
Bleeding during pregnancy (yes vs no)	5.3 (4.5 to 6.0)	5.0 (4.6 to 5.3)	0.3 (-0.5 to 1.1)	1.07 (0.90 to 1.26)
Placental abruption (yes vs no)	0.4 (0.2 to 0.6)	0.6 (0.5 to 0.7)	-0.2 (-0.5 to 0.0)	0.62 (0.36 to 1.09)
Prelabor rupture of membranes (yes vs no)	2.6 (2.0 to 3.2)	2.8 (2.6 to 3.1)	-0.2 (-0.9 to 0.4)	0.91 (0.71 to 1.17)
Mode of delivery (vs unassisted vaginal)				
Instrumental vaginal	17.5 (15.9 to 19.1)	16.8 (16.1 to 17.4)	0.8 (-1.0 to 2.5)	1.06 (0.93 to 1.20)
Planned cesarean	13.6 (12.2 to 15.0)	12.5 (11.9 to 13.0)	1.1 (-0.5 to 2.6)	1.10 (0.96 to 1.27)
Emergency cesarean	23.5 (21.8 to 25.2)	22.9 (22.2 to 23.6)	0.6 (-1.3 to 2.5)	1.04 (0.93 to 1.15)
Induced delivery (yes vs no)	21.0 (19.5 to 22.5)	21.0 (20.4 to 21.6)	0.0 (-1.6 to 1.6)	1.00 (0.91 to 1.10)
Infant death within 0-27 days (yes vs no)	0.4 (0.1 to 0.8)	0.1 (0.1 to 0.1)	0.3 (0.0 to 0.7)	4.53 (1.80 to 11.35)
Gestational age (vs term)				
Moderately preterm (32-36 weeks)	5.5 (4.7 to 6.4)	6.2 (5.9 to 6.6)	-0.7 (-1.7 to 0.2)	0.88 (0.74 to 1.05)
Very preterm (<32 weeks)	1.4 (1.0 to 1.9)	1.5 (1.3 to 1.7)	-0.1 (-0.6 to 0.4)	0.94 (0.67 to 1.31)
Birth weight (<2500g vs ≥2500g)	5.4 (4.6 to 6.2)	5.7 (5.4 to 6.1)	-0.4 (-1.3 to 0.5)	0.93 (0.78 to 1.11)
BWGA percentile (vs 25.0-74.9 percentile)				
<10.0	26.2 (24.2 to 28.3)	24.0 (23.2 to 24.7)	2.3 (0.0 to 4.5)	1.13 (1.00 to 1.27)
10.0-24.9	30.3 (28.2 to 32.4)	27.7 (26.9 to 28.4)	2.7 (0.4 to 5.0)	1.14 (1.02 to 1.27)
75.0-89.9	16.4 (14.6 to 18.2)	17.3 (16.6 to 18.0)	-0.9 (-2.9 to 1.0)	0.94 (0.81 to 1.08)
≥90.0	10.5 (9.0 to 12.1)	11.1 (10.5 to 11.7)	-0.6 (-2.3 to 1.1)	0.94 (0.79 to 1.12)
Apgar score (<7 vs ≥7)	1.9 (1.4 to 2.4)	1.8 (1.6 to 1.9)	0.2 (-0.4 to 0.7)	1.09 (0.80 to 1.48)
Congenital malformation (vs none)				
Any	5.1 (4.2 to 5.9)	4.3 (4.0 to 4.6)	0.8 (-0.1 to 1.7)	1.20 (0.99 to 1.45)
Major	3.0 (2.4 to 3.7)	2.4 (2.2 to 2.6)	0.6 (-0.1 to 1.3)	1.26 (0.98 to 1.61)
Minor	2.2 (1.6 to 2.7)	2.0 (1.8 to 2.1)	0.2 (-0.4 to 0.8)	1.12 (0.84 to 1.49)
Sex of baby (boy vs girl)	49.7 (47.8 to 51.5)	52.0 (51.3 to 52.7)	-2.4 (-4.4 to -0.4)	0.91 (0.84 to 0.99)

Abbreviations: AAR, adjusted absolute risk; ARD, adjusted risk difference; BWGA, birth weight for gestational age; OR, odds ratio; RRR, relative risk ratio:
[a]Odds ratios for binary outcomes and relative risk ratios for categorical outcomes, adjusted for maternal age at delivery, delivery year, maternal country of birth, previous spontaneous abortion, previous extra-uterine pregnancy and total number of stimulations.

© 2022 Rodriguez-Wallberg KA, et al. *JAMA Pediatrics.*

eTable 6. Sensitivity Analysis of Table 2, Including Adjustments by Means of Multiple Imputation

	Model 1				Model 2			
	AAR, % (95% CI)		ARD, percentage points (95% CI)	RRR or OR (95% CI)[a]	AAR, % (95% CI)		ARD, percentage points (95% CI)	RRR or OR (95% CI)[a]
	DET	SET			DET	SET		
Gestational hypertension (yes vs no)	5.9 (5.2 to 6.6)	5.8 (5.5 to 6.1)	0.10 (-0.65 to 0.85)	1.02 (0.89 to 1.17)	5.8 (5.2 to 6.5)	5.8 (5.5 to 6.1)	0.02 (-0.72 to 0.75)	1.00 (0.87 to 1.15)
Preeclampsia (yes vs no)	3.8 (3.3 to 4.4)	3.9 (3.7 to 4.2)	-0.13 (-0.73 to 0.48)	0.97 (0.82 to 1.14)	3.8 (3.2 to 4.3)	3.9 (3.7 to 4.2)	-0.18 (-0.78 to 0.42)	0.95 (0.81 to 1.12)
Gestational diabetes (yes vs no)	1.9 (1.5 to 2.3)	2.1 (2.0 to 2.3)	-0.22 (-0.64 to 0.20)	0.90 (0.72 to 1.12)	1.9 (1.5 to 2.2)	2.2 (2.0 to 2.3)	-0.28 (-0.69 to 0.13)	0.87 (0.69 to 1.08)
Bleeding during pregnancy (yes vs no)	5.0 (4.4 to 5.6)	4.7 (4.5 to 4.9)	0.34 (-0.32 to 1.01)	1.08 (0.94 to 1.24)	5.0 (4.4 to 5.6)	4.7 (4.5 to 4.9)	0.33 (-0.34 to 0.99)	1.07 (0.93 to 1.24)
Placental abruption (yes vs no)	0.6 (0.4 to 0.8)	0.6 (0.5 to 0.7)	0.01 (-0.24 to 0.25)	1.01 (0.67 to 1.52)	0.6 (0.4 to 0.8)	0.6 (0.5 to 0.7)	0.00 (-0.24 to 0.25)	1.01 (0.67 to 1.52)
Prelabor rupture of membranes (yes vs no)	2.5 (2.0 to 2.9)	2.5 (2.3 to 2.7)	-0.02 (-0.52 to 0.48)	0.99 (0.80 to 1.22)	2.4 (2.0 to 2.9)	2.5 (2.3 to 2.7)	-0.03 (-0.53 to 0.46)	0.99 (0.80 to 1.21)
Mode of delivery (vs unassisted vaginal)								
Instrumental vaginal	12.9 (11.8 to 14.1)	12.3 (11.8 to 12.7)	0.67 (-0.56 to 1.91)	1.07 (0.95 to 1.20)	12.9 (11.8 to 14.1)	12.3 (11.8 to 12.7)	0.68 (-0.56 to 1.91)	1.07 (0.95 to 1.20)
Planned cesarean	15.1 (13.9 to 16.2)	14.1 (13.7 to 14.6)	0.94 (-0.32 to 2.20)	1.08 (0.98 to 1.20)	15.0 (13.8 to 16.1)	14.1 (13.7 to 14.6)	0.86 (-0.40 to 2.11)	1.07 (0.97 to 1.19)
Emergency cesarean	19.5 (18.2 to 20.8)	18.6 (18.1 to 19.1)	0.86 (-0.53 to 2.25)	1.06 (0.97 to 1.16)	19.3 (18.1 to 20.6)	18.7 (18.2 to 19.1)	0.66 (-0.72 to 2.03)	1.05 (0.95 to 1.15)
Induced delivery (yes vs no)	19.5 (18.3 to 20.6)	19.1 (18.6 to 19.5)	0.41 (-0.85 to 1.68)	1.03 (0.95 to 1.11)	19.3 (18.2 to 20.4)	19.1 (18.6 to 19.5)	0.23 (-1.02 to 1.48)	1.02 (0.94 to 1.10)
Infant death within 0-27 days (yes vs no)	0.3 (0.1 to 0.5)	0.1 (0.1 to 0.1)	0.19 (-0.01 to 0.38)	2.69 (1.29 to 5.63)	0.3 (0.1 to 0.5)	0.1 (0.1 to 0.1)	0.18 (-0.01 to 0.37)	2.64 (1.26 to 5.52)
Gestational age (vs term)								
Moderately preterm (32-36 weeks)	5.3 (4.6 to 5.9)	5.8 (5.5 to 6.0)	-0.53 (-1.26 to 0.20)	0.90 (0.78 to 1.04)	5.2 (4.6 to 5.9)	5.8 (5.5 to 6.1)	-0.55 (-1.28 to 0.17)	0.90 (0.78 to 1.04)
Very preterm (<32 weeks)	1.4 (1.1 to 1.8)	1.2 (1.1 to 1.4)	0.20 (-0.19 to 0.60)	1.17 (0.88 to 1.55)	1.4 (1.1 to 1.8)	1.2 (1.1 to 1.4)	0.19 (-0.20 to 0.59)	1.16 (0.87 to 1.54)
Birth weight (<2500g vs ≥2500g)	4.8 (4.1 to 5.4)	4.8 (4.6 to 5.1)	-0.06 (-0.75 to 0.63)	0.99 (0.85 to 1.15)	4.7 (4.1 to 5.4)	4.8 (4.6 to 5.1)	-0.08 (-0.76 to 0.61)	0.98 (0.84 to 1.15)
BWGA percentile (vs 25.0-74.9 percentile)								
<10.0	21.9 (20.3 to 23.4)	20.7 (20.1 to 21.2)	1.24 (-0.45 to 2.93)	1.08 (0.97 to 1.19)	21.9 (20.4 to 23.5)	20.6 (20.1 to 21.2)	1.28 (-0.41 to 2.97)	1.08 (0.98 to 1.20)

© 2022 Rodriguez-Wallberg KA, et al. *JAMA Pediatrics.*

10.0-24.9	26.8 (25.2 to 28.4)	25.5 (24.9 to 26.1)	1.28 (-0.48 to 3.04)	1.07 (0.98 to 1.17)	26.9 (25.3 to 28.5)	25.5 (24.9 to 26.1)	1.37 (-0.39 to 3.13)	1.07 (0.98 to 1.18)
75.0-89.9	18.3 (16.8 to 19.7)	19.8 (19.3 to 20.4)	-1.55 (-3.14 to 0.04)	0.90 (0.81 to 1.00)	18.2 (16.7 to 19.6)	19.9 (19.3 to 20.4)	-1.69 (-3.27 to -0.11)	0.89 (0.80 to 1.00)
≥90.0	11.8 (10.5 to 13.0)	14.2 (13.7 to 14.7)	-2.44 (-3.80 to -1.09)	0.80 (0.71 to 0.91)	11.6 (10.4 to 12.8)	14.2 (13.7 to 14.7)	-2.61 (-3.95 to -1.28)	0.79 (0.69 to 0.90)
Apgar score (<7 vs ≥7)	1.6 (1.2 to 1.9)	1.6 (1.4 to 1.7)	0.01 (-0.40 to 0.42)	1.01 (0.77 to 1.31)	1.6 (1.2 to 1.9)	1.6 (1.4 to 1.7)	-0.00 (-0.41 to 0.40)	1.00 (0.76 to 1.30)
Congenital malformation (vs none)								
Any	4.5 (3.9 to 5.1)	4.0 (3.8 to 4.2)	0.46 (-0.21 to 1.12)	1.12 (0.96 to 1.31)	4.5 (3.9 to 5.1)	4.0 (3.8 to 4.2)	0.46 (-0.21 to 1.12)	1.12 (0.96 to 1.31)
Major	2.8 (2.3 to 3.2)	2.3 (2.1 to 2.5)	0.45 (-0.09 to 0.98)	1.20 (0.98 to 1.47)	2.8 (2.3 to 3.2)	2.3 (2.1 to 2.5)	0.44 (-0.09 to 0.97)	1.20 (0.98 to 1.47)
Minor	1.8 (1.4 to 2.2)	1.8 (1.6 to 1.9)	0.02 (-0.41 to 0.46)	1.01 (0.79 to 1.30)	1.8 (1.4 to 2.2)	1.8 (1.6 to 1.9)	0.03 (-0.41 to 0.46)	1.02 (0.79 to 1.30)
Sex of baby (boy vs girl)	49.9 (48.4 to 51.4)	51.6 (51.1 to 52.2)	-1.74 (-3.36 to -0.12)	0.93 (0.87 to 1.00)	49.9 (48.4 to 51.4)	51.7 (51.1 to 52.2)	-1.75 (-3.37 to -0.13)	0.93 (0.87 to 0.99)

Abbreviations: AAR, adjusted absolute risk; ARD, adjusted risk difference; BWGA, birth weight for gestational age; OR, odds ratio; RRR, relative risk ratio.

[a]Relative risk denotes odds ratios for binary outcomes and relative risk ratios for categorical outcomes. Model 1 adjusted for maternal age at delivery, birth order, delivery year, maternal country of birth, previous spontaneous abortion, previous extra-uterine pregnancy, total number of stimulations and fertilization method. Model 2 adjusted similarly to as in Model 1, with additional adjustments for maternal education, body-mass index (BMI) and smoking during pregnancy.

Multivariate imputation by chained equations (MICE) were used for handling missing values of for fertilization method, maternal education, BMI, smoking in 7% incomplete cases. Smoking was imputed using logistic regression and multinomial logistic regression was used for imputing each of the other covariates. All covariates were assumed to be missing at random given the predictors in the imputation models; maternal age, year of delivery, birth order, country of birth, previous spontaneous abortion or extra-uterine pregnancy, total number of stimulation cycles, gestational hypertension or preeclampsia, placental abruption, pre-labor rupture of membranes, pregnancy length, mode of delivery, pregnancy bleeding, malformations (none, major, minor), sex of child, as well as the other imputed variables. Ten cycles of chained equations were used. The same imputed datasets were used for all outcomes, and results from 50 imputation cycles were combined by Rubin's rules. Adjusted absolute risks and absolute risk differences predicted using the postestimation command `mimrgns` in Stata 17.0/BE.

© 2022 Rodriguez-Wallberg KA, et al. *JAMA Pediatrics.*
